# Genome-Wide Characterization of *Solanum tuberosum* CCoAOMT Gene Family and Identification of StCCoAOMT Genes Involved in Anthocyanin Biosynthesis

**DOI:** 10.3390/genes15111466

**Published:** 2024-11-13

**Authors:** Yaxuan Peng, Suao Sheng, Tongtong Wang, Jiafeng Song, Daijuan Wang, Yixuan Zhang, Jielan Cheng, Tingting Zheng, Zhaoyan Lv, Xiaobiao Zhu, Hualan Hou

**Affiliations:** School of Horticulture, Anhui Agricultural University, Hefei 230036, China

**Keywords:** caffeoyl-CoA-O methyltransferase, expression pattern, functional analysis, *StCCoAOMT10*, subcellular localization

## Abstract

Background: The caffeoyl-CoA-O methyltransferase (CCoAOMT) family plays essential roles in the methylation of various secondary metabolites, including anthocyanins. Despite the wide identification of the CCoAOMT family in plants, the characterization and function of CCoAOMT protein members in *Solanum tuberosum* remain poorly understood. Methods and Results: In this study, a total of 12 StCCoAOMT members were identified in the genome of *S. tuberosum* using the Blastp and HMM search and were unevenly located on eight chromosomes. Collinearity analysis revealed that four tandem duplicated gene pairs and two segmental duplicated gene pairs existed in the *S. tuberosum* genome, demonstrating that duplication events play a key role in the expansion of the CCoAOMT family. All StCCoAOMTs were clustered into group I and group II based on phylogenetic analysis, which was further verified by the conserved motifs and gene structures analysis. The *cis*-acting elements analysis illustrated that StCCoAOMTs might be important for photosynthesis, hormone responses, and abiotic stress. Expression analysis demonstrated that *StCCoAOMT* genes have diverse transcript levels in various tissues and that *StCCoAOMT10* was significantly expressed in purple potatoes with abundant anthocyanin content according to RNA-seq data and qRT-PCR assays. In addition, the subcellular localization assay validated that the StCCoAOMT10 protein was mainly localized in the cytoplasm and nucleus. Conclusions: These results will be of great importance for a better understanding of the features of CCoAOMT family members, especially of the candidate genes involved in the methylation of anthocyanins in *S. tuberosum*, and also for improving the nutritional quality of *S. tuberosum*.

## 1. Introduction

*S. tuberosum*, as the fourth largest staple crop in the world, followed by *Oryza sativa*, *Zea mays*, and *Triticum aestivum*, plays an important role in ensuring global food security [1,2]. Potato plants contain a variety of secondary metabolites, including lignin and flavonoids. Lignin mainly synthesizes in secondary thickening cells and plays a mechanical support role for plant cells and tissues, contributing to the transportation of nutrients and water [3]. Lignin has been widely studied in a range of plants because it is a crucial product of the phenylalanine metabolism pathway. Flavonoids are a class of polyphenol compounds in plants that play various roles in plant biological processes such as organ development, plant coloration, abiotic stress response, and hormone transport [4,5]. For example, shade plants, which are rich in kaempferol and/or apigenin derivatives, have the characteristics of large leaf lamina, a long internode, and decreased leaf thickness [6]. Quercetin 3-O-rutinoside (rutin) may enhance membrane rigidity by interacting with the polar head of phospholipids at the lipid–water interface, thereby protecting membranes from oxidative damage [7]. The flavonol compounds play specific roles in UVB tolerance [8,9]. Furthermore, several studies have indicated that flavonoids are responsible for an array of therapeutic potential in humans, such as cancer, diabetes, and cardiovascular diseases [10]. For instance, quercetin and its derivatives were used for the treatment of osteoporosis due to their natural antioxidant properties [11,12]. Anthocyanins play important roles in repressing the reproduction of cell lines for human stomach cancer, prostate cancer, and erythrocyte leukemia [13].

Anthocyanins derived from the flavonoid pathway play essential roles in flower and fruit pigmentation, plant development, pollination and seed dispersal, herbivore and pathogen defense, and stress response [14,15]. For example, delphinidin-based derivatives, which were ubiquitously detected in the purple fruits of *Solanum lycopersicum*, *Solanum melongena*, and *Capsicum annuum*, are considered the main reason for the formation of fruit colors [16]. At present, large numbers of anthocyanins have been identified in plants, and six common anthocyanins widely exist in potato tubers, namely, pelargonidin, cyanidin, delphinidin, peonidin, malvidin, and petunidin. Among them, pelargonidin is the predominant anthocyanin in red potato tubers, while petunidin is the main anthocyanin in purple potato tubers [16,17,18]. The biosynthesis of anthocyanins includes acetylation, glycosylation, and O-methylation at different positions of the flavonoid skeleton (e.g., naringenin). Methylation modification is proposed to modify the water solubility of anthocyanins and improve their stability, thus significantly promoting the accumulation of color [19,20,21]. Anthocyanin metabolism is regulated by many factors, including genetic, developmental, and environmental conditions. In terms of genetic regulation, the induction or closing of anthocyanin biosynthesis depends on the transcript abundance of the regulatory and structural genes in plants [16]. Among them, structural genes encode the enzymes that catalyze each reaction step, including *CHS*, *CHI*, *F3H*, *F3′H*, *F3′5′H*, *DFR*, *ANS*, *3GT*, *AT*, and *OMT*, while regulatory genes encode transcription factors controlling the expression of the structural genes, such as MYB, basic helix–loop–helix (bHLH), and the WD40 repeat protein [22,23].

O-methyltransferases (OMTs) are various groups of multifunctional enzymes that play a crucial role in regulating several secondary metabolic processes, including lignin and flavonoid biosynthesis [24,25]. Plant OMTs were divided into two primary families according to their enzymatic properties and molecular weight: the caffeic acid O-methyltransferase (COMT) family with subunit sizes of 38–42 kDa and the Mg^2+^-dependent caffeoyl coenzyme An O-methyltransferase (CCoAOMT) family with low subunit sizes of 26–30 kDa [26,27]. Numerous studies have shown that the COMT subfamily plays important roles in lignin, melatonin, and flavonoid biosynthesis [28,29,30,31,32,33]. For instance, the *CitOMT2* gene was verified as flavonoid 8-O-methyltransferase in vitro [34]. The overexpression of *COMT1* contributes to melatonin biosynthesis in tomato plants [35]. The CCoAOMT family acts as a key enzyme that participates in lignin biosynthesis and plays a vital role in converting the Caffeoyl-CoA into Feruloyl-CoA in plants [36]. CCoAOMT proteins possess a complete AdoMet MTAses domain and comprise eight conserved motifs designated as A to H. Among them, motifs A (LVKVGGLIG), B (VAPPDAPLRKY), and C (ALAVDPRIEICM) are general characterizations that exist in all OMTs, whereas motifs D (TSVYPREPEPMKELRELT), E (KLINAKNTMEI), F (PVIQKAGVAHKIEF), G (DFIFVDADKDNY), and H (GDGITLCRR) are specific to CCoAOMT. To date, CCoAOMT family members have been widely identified in diverse species, for example, a total of 6 *CCoAOMT* genes were presented in the *Populus trichocarpa* genome [37], 7 in *Sorghum bicolo*r [38], 21 in *T. aestivum* [39], and 10 in *Vitis vinifera* [25]. However, to the best of our knowledge, a comprehensive survey of StCCoAOMT gene family members has not been conducted and little functional information exists regarding CCoAOMT in potatoes.

In addition to lignin biosynthesis, CCoAOMT family members have also been reported to be involved in flavonoid biosynthesis. The first *CCoAOMT* gene associated with the methylation of flavonoids, named *PFOMT*, was identified from *Mesembryanthemum crystallinum*, suggesting that CCoAOMT members participated in the biosynthesis of flavonoids [40]. Unlike other CCoAOMTs that participate in monolignol biosynthesis, PFOMTs accept a wide array of compounds with a vicinal dihydroxyl structure as substrates and exhibit a preference for phenylpropanoids like flavonols and caffeic acid esters [40]. Thus, the CCoAOMT family was further classified into two clades: true CCoAOMT and CCoAOMT-like (PFOMT). Generally, the true CCoAOMT members are involved in lignin biosynthesis in vivo through methylating caffeoyl-CoA, and some members are capable of catalyzing the methylation of flavonoids in vitro [41,42,43], while the catalytic activity of PFOMT members is mainly manifested in the methylation of flavonols and anthocyanins. Currently, several *CCoAOMT*-like genes associated with flavonoid and anthocyanin methylation have been reported, such as *VvAOMT* from *V. vinifera* [20], *SlAnthOMT* from *S. lycopersicum* [44], *PpAOMT2* from *Prunus persica* [45], and *CdFOMT5* from *Citrus depressa* [46]. Nevertheless, the potential functions of CCoAOMT family genes on anthocyanin biosynthesis remain unclear in potatoes.

To systematically characterize the potato CCoAOMT gene family, bioinformatics analysis was employed to identify and analyze the CCoAOMT family members, including the chromosome location, duplication event, evolutionary relationship, gene structure, and *cis*-regulatory element. Furthermore, the candidate StCCoAOMT gene involved in anthocyanin biosynthesis was identified by RNA-seq and qRT-PCR analysis. This study is conducive to the acknowledgment of *CCoAOMT* genes and provides a foundation for future research into the function of these genes in potatoes.

## 2. Materials and Methods

### 2.1. Plant Materials and Growth Conditions

The plant materials K149-W, K149-Y, and K149-P, representing the white, yellow, and purple tuber flesh, respectively, were introduced from the U.S. National Plant Germplasm System (https://npgsweb.ars-grin.gov/gringlobal/search, accessed on 30 May 2021) and cultivated in a glass greenhouse in Anhui Agricultural University. The samples of tuber flesh were collected from potato K149 lines for expression pattern analysis.

*Nicotiana benthamiana* was planted in plastic pots containing peat soil and vermiculite (1:1) and grown in a climate room, in which the conditions were as follows: 16 h light (23 °C)/8 h dark (18 °C) and relative humidity of 40–60%. Four-week-old *N. benthamiana* was used for the subcellular localization assay.

### 2.2. Identification of S. tuberosum CCoAOMT Genes

The HMM profile of the CCoAOMT conserved domain (PF01596) was retrieved from the InterPro database (https://www.ebi.ac.uk/interpro/, accessed on 1 July 2024). The AtCCoAOMT protein sequences of Arabidopsis were downloaded from the TAIR database (https://www.arabidopsis.org/, accessed on 1 July 2024). The candidate StCCoAOMT protein sequences were identified by HMMER 3.0 software using the HMM profile and Blastp procedure using AtCCoAOMT proteins (e-value < 1 × 10^−20^). The common proteins in both HMM and Blastp search were chosen, and the redundant proteins were removed. Subsequently, the candidate *StCCoAOMT* genes were further verified using SMART (https://smart.embl.de/, accessed on 5 July 2024), InterPro (https://www.ebi.ac.uk/interpro/, accessed on 5 July 2024), and NCBI CD-Search (https://www.ncbi.nlm.nih.gov/Structure/cdd/wrpsb.cgi, accessed on 5 July 2024) websites [47]. The physical and chemical properties of StCCoAOMT proteins, including the number of amino acids, molecular weight (MW), theoretical pI, grand average of hydropathicity (GRAVY), and instability index (II), were analyzed using the ExPASy online website (https://web.expasy.org/protparam/, accessed on 10 July 2024) [48]. The subcellular localization of StCCoAOMT proteins was predicted by WoLF PSORT (https://wolfpsort.hgc.jp/, accessed on 10 July 2024).

### 2.3. Chromosomal Distribution and Collinearity Analysis

The information of *StCCoAOMTs* mapped on the chromosomes was extracted from the genome sequence and GFF3 file of ‘DM’ in Spud DB (http://spuddb.uga.edu/, accessed on 12 July 2024). The duplication event of *CCoAOMT* genes within the ‘DM’ genome and the collinearity analysis of the ‘DM’ cultivar associated with *Arabidopsis thaliana*, *Camellia sinensis*, and *S. lycopersicum* genomes were carried out and visualized by TBtools software (v 2.136) [49].

### 2.4. Phylogenetic Tree, Conserved Motif and Gene Structure Analysis

The CCoAOMT protein sequences of *A. thaliana*, *O. sativa*, and *C. sinensis* were downloaded from TAIR, Phytozome, and NCBI databases, respectively. An unrooted phylogenetic tree was constructed using MEGA 11.0 using the maximum likelihood method with 1000 replicate bootstraps and optimized using the iTOL website (Interactive Tree of Life, https://itol.embl.de, accessed on 20 July 2024) [50]. The CCoAOMT protein sequences of *S. tuberosum* were submitted to the online website MEME (https://meme-suite.org/meme/tools/meme, accessed on 22 July 2024) for conserved motif analysis [51]. The exon–intron structure was analyzed using the GSDS website (Gene Structure Display Server, https://gsds.gao-lab.org/, accessed on 22 July 2024). The integrated graph of the evolutionary tree, conserved motifs, and exon–intron structure was visualized by TBtools software [49].

### 2.5. Cis-Regulatory Elements Analysis of the StCCoAOMT Promoters

The 2000 bp promoter sequence of *StCCoAOMT* genes was extracted from the *S. tuberosum* genomic file (.gff) and then submitted to the PlantCARE website (http://bioinformatics.psb.ugent.be/webtools/plantcare/html, accessed on 24 July 2024) to analyze the *cis*-acting regulatory elements [52]. The heatmap of *cis*-elements in the promoter regions was visualized by TBtools software [49].

### 2.6. Total RNA Extraction and qRT–PCR Analysis

Total RNA was extracted from potato K149 lines using the RNAprep Pure Plant Plus Kit (TIANGEN, Beijing, China). The first strand of cDNA was synthesized by the reverse transcription of 1 μg of RNA utilizing the PrimeScript™ first-strand cDNA synthesis kit (TaKaRa, Kyoto, Japan). The qRT-PCR assay was carried out using the SYBR Premix Ex Taq II Kit (Takara, Kyoto, Japan). Each 10 µL of the reaction system consisted of 0.5 µL of cDNA, 0.25 µL of the forward primer, 0.25 µL of the reverse primer, 5 µL of TB Green™ Premix Ex Taq™ II, and 4 µL of RNase-free H_2_O. The reaction procedure was as follows: 95 °C for 30 s; 95 °C for 10 s, 60 °C for 20 s, 72 °C for 30 s, and 40 cycles. The relative expression was calculated by the 2^−ΔΔCt^ method. The *StActin* was selected as the housekeeping gene. The gene-specific primers were designed by the NCBI website and are listed in Table S1.

### 2.7. Gene Cloning and Vector Construction

The cDNA sample of K149-P tuber flesh was utilized as the template for PCR amplification. The full coding sequence of the *StCCoAOMT10* gene was cloned using PrimeSTAR^®^ GXL DNA Polymerase and gene-specific primers. The amplified target fragment was integrated with the linearized pRI101 vector digested with the *Sal* I and *Bam*H I enzymes using the homologous recombination method to generate overexpression vector 35S::StCCoAOMT10-GFP and then transform the positive plasmids into *Agrobacterium tumefaciens* strain GV3101.

### 2.8. Subcellular Localization of StCCoAOMT10 in Tobacco

The positive Agrobacterium clones containing 35S::StCCoAOMT10-GFP and nuclear-localized marker (H_2_B-mCherry) were cultured in the liquid Luria–Bertani (LB) medium at 28 °C and resuspended in the infection buffer (10 mM MES, 10 mM MgCl2, 150 μM acetosyringone) to OD600 = 0.8, respectively. Four-week-old *N. benthamiana* leaves were used for the co-injection of the above two suspensions, and the empty vector (35S::GFP) was used as a control. After 48 h, the Agrobacterium-inoculated tobacco leaves were cut into 1 × 1 cm sizes for the preparation of samples. The signals of the green fluorescent protein (GFP) and red fluorescent protein (RFP, H_2_B-mCherry) of leaves were observed and photographed by a laser scanning confocal microscope (Leica STELLARIS 5, Mannheim, Germany) [53]. The excitation wavelengths of 488 nm and 580 nm were used for GFP and RFP observation, respectively.

## 3. Results

### 3.1. Genome-Wide Identification and Physicochemical Analysis of the CCoAOMT Family in S. tuberosum

The StCCoAOMT family member was identified in the *S. tuberosum* (cultivar ‘DM’) genome through BLASTP and HMMER searches. Initially, we obtained 19 putative *StCCoAOMT* genes with the BLASTP search and 18 putative *StCCoAOMT* genes with the HMMER search. Subsequently, 13 unique genes were filtered by CDD, SMART, and InterProScan analyses to remove the false-positive sequences. Eventually, 12 *CCoAOMT* genes were identified in the potato genome and labeled as *StCCoAOMT1–StCCoAOMT12* according to the order of localization on the chromosomes (Table 1).

The coding sequence (CDS) lengths of *StCCoAOMT* genes ranged from 369 to 1323 nucleotides. The length of StCCoAOMT protein sequences varied from 123 to 441 amino acids. The StCCoAOMT proteins had a maximum and minimum molecular weight of 13.72 kilodaltons (kDa) (StCCoAOMT12) and 49.72 kDa (StCCoAOMT6), respectively, and the average molecular weight was 28.22 kDa. The proteins of StCCoAOMT genes had a theoretical pI spectrum of 4.93 (StCCoAOMT12) to 7.08 (StCCoAOMT9). About 58.33% of the StCCoAOMT proteins (7 StCCoAOMTs) were considered stable proteins. The prediction of the grand average of hydropathicity (GRAVY) values demonstrated that all StCCoAOMT proteins are hydrophilic (Table 1). In addition, the prediction of subcellular localization showed that approximately 66.67% of the StCCoAOMT proteins (eight) were located in the cytoplasm region, two in the cytoskeleton (StCCoAOMT4 and StCCoAOMT5), and two in the chloroplast (StCCoAOMT8 and StCCoAOMT9).

### 3.2. Chromosome Mapping of StCCoAOMT Genes

**Figure 1:**
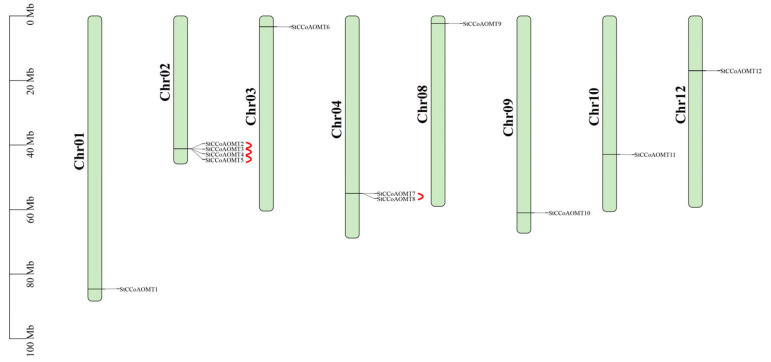
Distribution of *StCCoAOMT* genes in the potato genome.

All *StCCoAOMT* genes were physically mapped onto eight chromosomes. The *StCCoAOMT* genes were not evenly distributed on eight chromosomes in the potato but were densely located in two chromosome regions. Among them, chromosome 2 was the main distribution site, accounting for 33.33% of the total *StCCoAOMT* genes, followed by chromosome 4, accounting for 16.67% of the total *StCCoAOMT* genes. Only one StCCoAOMT member was distributed in chromosomes 1, 3, 8, 9, 10, and 12 (Figure 1). There was no correlation between the number of *StCCoAOMT* genes and the chromosome length. Additionally, an obvious tandem repeat gene cluster was found on chromosome 2. Duplication analysis exhibited that a total of four tandemly duplicated gene (TDG) pairs with six *StCCoAOMT* genes were found in potato chromosomes (Figure 1, Table S2).

### 3.3. Evolutionary Analysis of StCCoAOMT Genes

**Figure 2:**
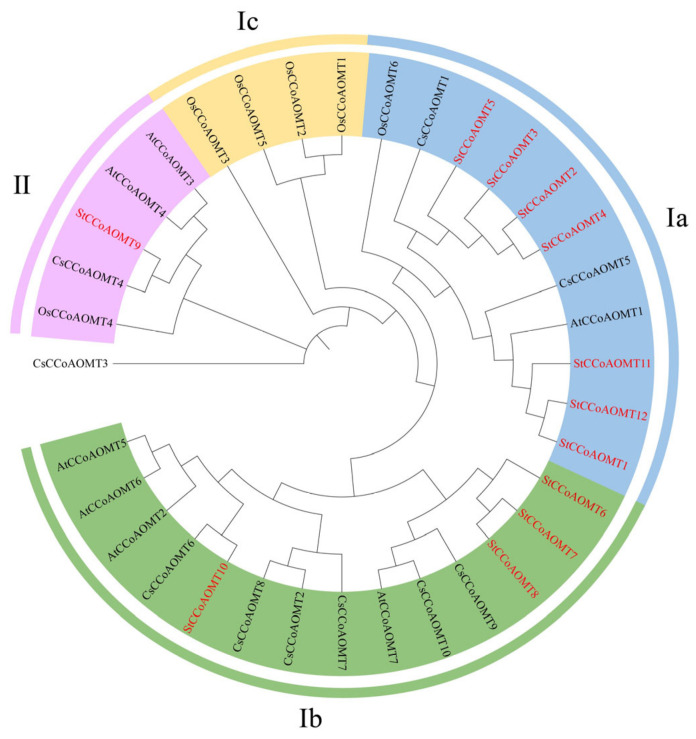
Phylogenetic relationship of CCoAOMTs from S. *tuberosum* and other species. At: *A. thaliana*, Os: *O. sativa*, Cs: *C. sinensis*, St: S. *tuberosum*. The four groups were differentiated with different colors, and CCoAOMTs from potatoes are highlighted with red font.

To elucidate the phylogenetic relationship of the CCoAOMT gene family, an unrooted evolutionary tree was constructed based on 35 CCoAOMT proteins, including 7 members from *A. thaliana*, 6 members from *O. sativa*, and 10 members from *C. sinensis* (Figure 2, Table S3). The phylogenetic analysis illustrated that all CCoAOMT proteins were categorized into two clades: group I and group II. Among them, group I contained three subbranches: Ia, Ib, and Ic. StCCoAOMT1~StCCoAOMT5, StCCoAOMT11, and StCCoAOMT12 were clustered with AtCCoAOMT1, CsCCoAOMT1, CsCCoAOMT5, and OsCCoAOMT6 into subbranch Ia, which was verified to be involved in lignin biosynthesis. Four StCCoAOMTs (StCCoAOMT6, StCCoAOMT7, StCCoAOMT8, and StCCoAOMT10) were grouped in subbranch Ib. Except for the CCoAOMTs of *O. sativa*, CCoAOMTs of the other species were not classified into Ic branch, suggesting that subgroup Ic might only exist in monocotyledons. Group II included StCCoAOMT9, CsCCoAOMT4, OsCCoAOMT4, AtCCoAOMT3, and AtCCoAOMT4 and exhibited a distant evolutionary relationship with Group I.

### 3.4. Intraspecific and Interspecific Collinearity Analysis of CCoAOMT Genes

**Figure 3:**
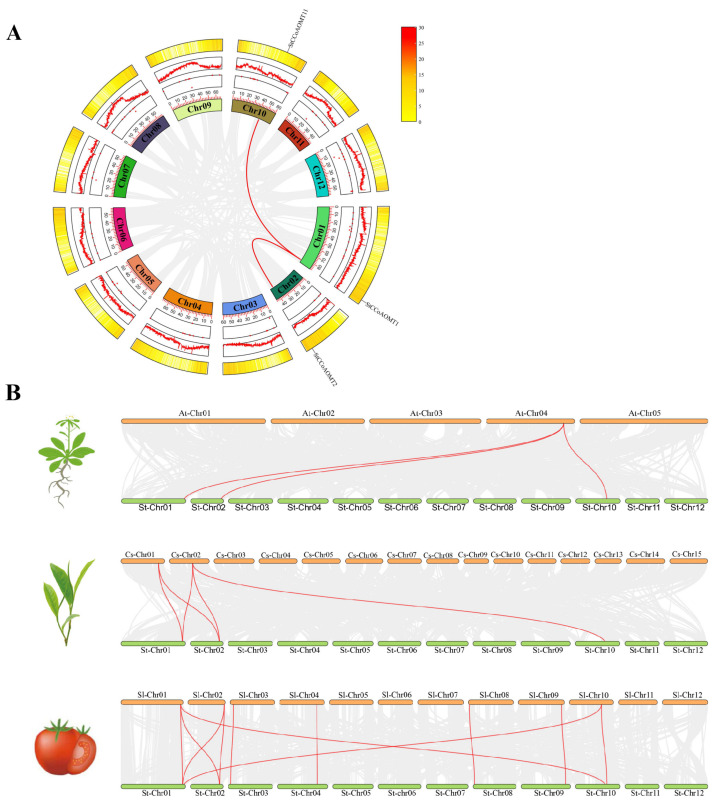
Collinearity analysis of *S. tuberosum* and different species. (**A**) Intraspecific collinearity analysis of *StCCoAOMT* genes. (**B**) Evolutionary relationship analysis between *S. tuberosum* to *A. thaliana*, *C. sinensis*, and *S. lycopersicum*. Gray lines represent all synteny blocks identified between the genomes of different species, and red lines represent the gene pairs with duplicated events.

Duplication events have vital biological significance in the plant kingdom. To investigate the evolutionary relationships among *StCCoAOMT* genes, the collinearity within the StCCoAOMT family was analyzed. The result indicated that only two homologous pairs (*StCCoAOMT1*/*StCCoAOMT2* and *StCCoAOMT1*/*StCCoAOMT11*) were identified in the StCCoAOMT family (Figure 3A). In addition, the ratios of non-synonymous (Ka) and synonymous (Ks) substitution (Ka/Ks) of segmentally duplicated gene pairs were lower than 1, indicating that the *StCCoAOMT* gene had undergone purifying selection.

To further explore the evolutionary relationships of CCoAOMT family members, the interspecies comparative syntenic maps concerning *A. thaliana*, *C. sinensis*, and *S. lycopersicum* were constructed. Interspecific collinearity analysis demonstrated that three *StCCoAOMT* genes displayed syntenic relationships with *AtCCoAOMTs* (*StCCoAOMT1*/*AtCCoAOMT1*, *StCCoAOMT2*/*AtCCoAOMT1*, and *StCCoAOMT11*/*AtCCoAOMT1*). A total of 5 pairs of collinear genes were found between *S. tuberosum* and *C. sinensis*, and 11 pairs of collinear genes were found between *S. tuberosum* and *S. lycopersicum*, with higher homology observed between *S. tuberosum* and *S. lycopersicum* (Figure 3B, Table S4), illustrating *S. tuberosum* had a closer evolutionary relationship with *S. lycopersicum*.

### 3.5. Conserved Motif Composition and Gene Structure of StCCoAOMT Family Members

**Figure 4:**
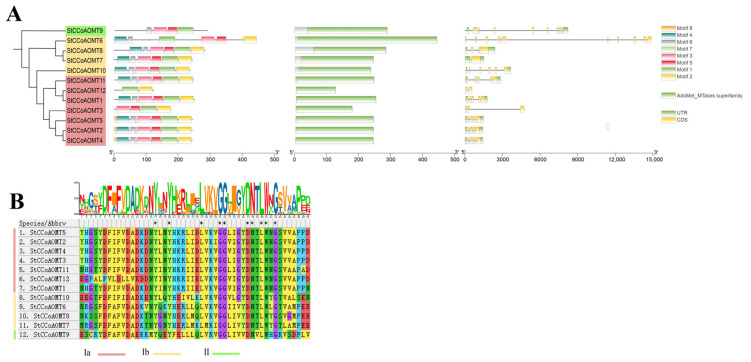
Conserved domain and gene structure analysis. (**A**) The phylogenetic relationship, conserved motifs, domain distribution, and exon–intron structures of the StCCoAOMTs. (**B**) The HHM logos of the conserved motif 1 in all StCCoAOMT proteins. The asterisk “*” indicates a highly conserved protein.

To gain further insight into the structural characteristics of StCCoAOMT proteins, the distribution of conserved motifs was predicted using MEME. A total of eight conserved motifs were identified and designated as motifs 1–8 (Figure 4, Figure S1). Members of the StCCoAOMT family had a relatively conserved motif distribution comprising only one AdoMet_Mtases superfamily protein structural domain. The number of conserved motifs varied from two to eight among different StCCoAOMT members and shared similar motif distributions within the same subclass, which is consistent with the phylogenetic tree result. In subgroup Ia, with the exception of StCCoAOMT3 and StCCoAOMT12, all StCCoAOMT proteins contain motifs 1–7. Among them, StCCoAOMT2, StCCoAOMT4, and StCCoAOMT5 contain all motifs. The StCCoAOMT members in subgroup Ib possess motifs 1–6, whereas group II member StCCoAOMT9 only contains motifs 1, 3, 5, and 6. Motif 1 was generally presented in all twelve StCCoAOMT members, demonstrating that it may be critical for the conserved function of StCCoAOMT proteins.

In addition, the exon–intron arrangement patterns of StCCoAOMT family members were analyzed. As illustrated in Figure 4A, the exon numbers of *StCCoAOMT* genes ranged from 2 to 10. The majority of StCCoAOMT members (*StCCoAOMT1*, *StCCoAOMT2*, *StCCoAOMT4*, *StCCoAOMT5*, *StCCoAOMT10*, and *StCCoAOMT11*) possess five exons and four introns, *StCCoAOMT3* and *StCCoAOMT8* have three exons and two introns, and the gene structure of *StCCoAOMT6* and *StCCoAOMT9* differed prominently from that of other members featuring ten and nine exons, respectively. Specifically, *StCCoAOMT7* and *StCCoAOMT12* exhibit a distinct pattern with only two exons. These results uncovered that StCCoAOMTs have diverse structural patterns based on their features and that members categorized into the same subgroup possess similar conserved motifs and exon–intron structures. Thus, we speculated that *StCCoAOMT* genes within the same clade may share functional similarities.

### 3.6. Cis-Regulatory Elements Analysis of StCCoAOMT Genes

**Figure 5:**
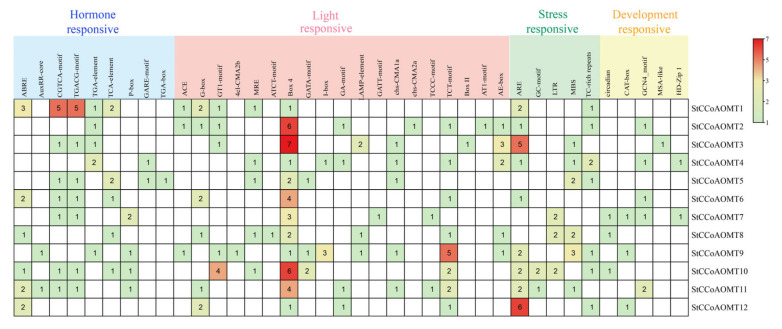
Analysis of *cis*-regulatory elements in the promoter region of *StCCoAOMT* genes.

To investigate the potential function of *StCCoAOMT* genes, the *cis*-elements of the 2000 bp promoter sequence were analyzed. Based on their potential functions, these elements can be classified into four types: plant growth and development elements, light-responsive elements, stress-responsive elements (biotic and abiotic stress), and phytohormone-responsive elements. The light-responsive *cis*-acting elements were widely distributed in promoter regions of all *StCCoAOMT* genes, indicating that the transcription of *StCCoAOMT* was regulated by light signals. Furthermore, 56 plant hormone response elements were found in *StCCoAOMT* genes, containing abscisic acid-(ABA), auxin-(IAA), methyl jasmonate-(MeJA), gibberellin-, and salicylic acid-responsive (SA) elements. Except for *StCCoAOMT2* and *StCCoAOMT12*, the promoter sequence of all *StCCoAOMT* genes had several types of hormone-responsive elements, suggesting *StCCoAOMT* genes are induced by various hormones. Among them, *StCCoAOMT1* had 16 phytohormone-responsive elements in the promoter region, and *StCCoAOMT1*, *StCCoAOMT5*, *StCCoAOMT10,* and *StCCoAOMT11* had 5 types of hormone-responsive elements (Figure 5). In addition, a variety of stress-related *cis*-regulatory elements were found in the promoter regions. For example, the low-temperature (LTR) element was observed in the promoter regions of *StCCoAOMT7*, *StCCoAOMT8*, and *StCCoAOMT10*, and the MYB-binding sites associated with the drought response were discovered in the upstream regions of *StCCoAOMT5*, *StCCoAOMT8*, and *StCCoAOMT9* genes.

### 3.7. Expression Pattern of StCCoAOMT Genes in Various Tissues

**Figure 6:**
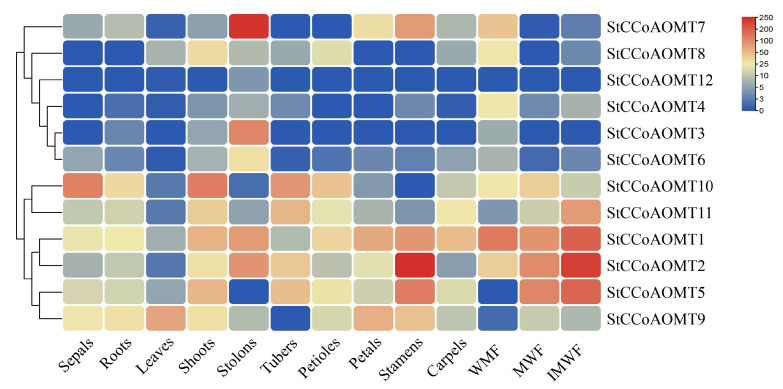
The tissue-specific expression patterns of *StCCoAOMT* genes at different developmental stages of potato. Transcript levels of *StCCoAOMT* gene in sepals, roots, leaves, shoots, stolons, tubers, petioles, petals, stamens, carpels, whole mature flower (WMF), mature whole fruit (MWF), and immature whole fruit (IMWF).

To explore the function of the *StCCoAOMT* genes in potatoes, the expression patterns of *StCCoAOMT* genes in various tissues were analyzed. As shown in Figure 6, *StCCoAOMT* gene expressions were diverse among the sepals, roots, leaves, shoots, stolons, petioles, tubers, petals, stamens, carpels, whole mature flower (WMF), mature whole fruit (MWF), and immature whole fruit (IMWF). *StCCoAOMT1* was widely expressed in different tissues, especially in the mature flower and immature fruit, implying that it may play an important role in different growth and development stages. *StCCoAOMT2* was highly expressed in the stamen, immature fruit, and mature fruit, speculating that *StCCoAOMT2* mainly functioned during fruit development. With the exception of stolons, *StCCoAOMT3* had low or no transcript level in all tissues. *StCCoAOMT7* has an extremely significant expression in stolons, while *StCCoAOMT9* has a relatively high expression level in leaves, petals, and stamens. *StCCoAOMT10* had high expression in sepals, shoots, and tubers. Except for *StCCoAOMT9*, the expression levels of all *StCCoAOMT* genes were generally low in leaves. In addition, some *StCCoAOMT* genes were not expressed or have low expression levels in various tissues, such as *StCCoAOMT4*, *StCCoAOMT6,* and *StCCoAOMT12*.

### 3.8. Identification of Candidate StCCoAOMT Gene Involved in Anthocyanin Biosynthesis

Methylation modification is an important step in the biosynthesis of anthocyanins. Given the wide participation of *CCoAOMT* genes in the biosynthesis of secondary metabolites, including flavonoids in plants, we speculated that StCCoAOMT members also play pivotal roles in anthocyanin synthesis in potatoes, thereby enhancing their chemical stability. Currently, the key *CCoAOMT* gene and its function in the methylation process of anthocyanins have not yet been systematically investigated and elucidated in potatoes.

**Figure 7:**
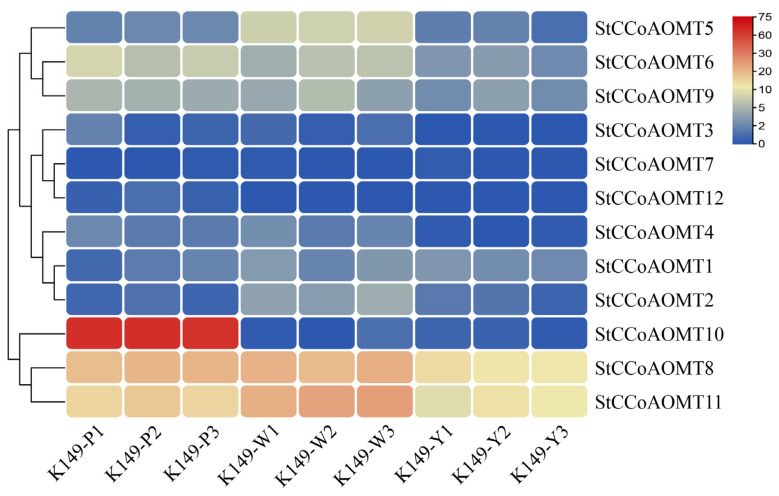
Identification of *StCCoAOMT* genes associated with anthocyanin biosynthesis.

**Figure 8:**
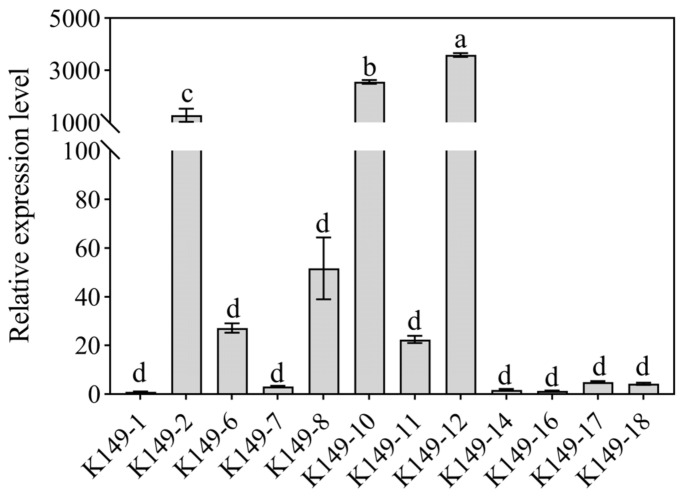
Expression levels of *StCCoAOMT10* gene in different K149 lines. The *X*-axis shows different potato K149 lines with different tuber flesh colors. White tuber flesh: K149-1/6/16/17; Yellow tuber flesh: K149-7/11/14/18; Purple tuber flesh: K149-2/8/10/12. The lowercase letters represent statistical significance (*p  *<  0.05).

To explore the potential members related to the formation of O-methylated flavonoids in potatoes, the expression patterns of StCCoAOMT members were analyzed in potato K149 lines with different colors. Expression level analysis discovered that *StCCoAOMT3*, *StCCoAOMT7,* and *StCCoAOMT12* were not expressed or had low expression levels in different lines (Figure 7). The transcript levels of *StCCoAOMT8* and *StCCoAOMT11* in white (K149-W) and purple (K149-P) tubers were relatively higher than those in yellow tubers (K149-Y), and the expression levels were the highest in white tubers. The expression abundance of *StCCoAOMT10* was significantly higher in the tuber-fleshes of the K149-P line with high anthocyanin content than that in the K149-W and K149-Y lines with lower anthocyanin content. In addition, RT-qPCR analysis further revealed that the transcript level of *StCCoAOMT10* gene in all K149 lines with purple tuber flesh (K149-2/8/10/12) was significantly higher than it in white (K149-1/6/16/17) and yellow tuber flesh (K149-7/11/14/18) (Figure 8). Therefore, the *StCCoAOMT10* was considered a key factor affecting the anthocyanin biosynthesis.

### 3.9. Subcellular Localization of the StCCoAOMT10 Protein

**Figure 9:**
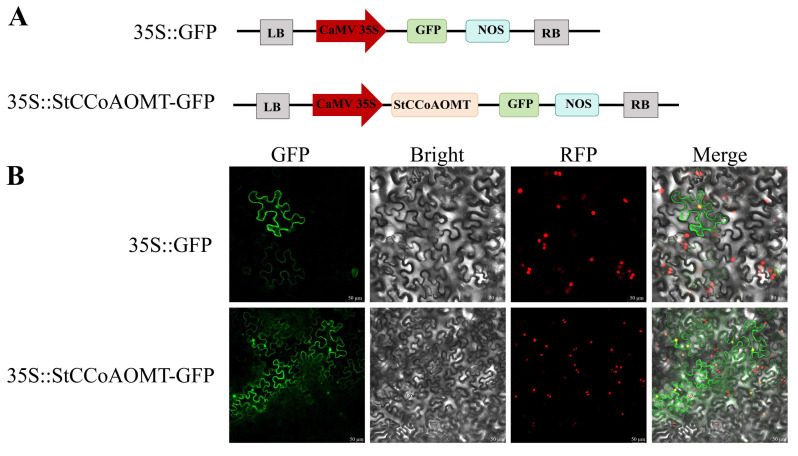
Subcellular localization of StCCoAOMT10 protein. (**A**) Illustration of empty vector (35S::GFP) and recombinant constructs (35S::StCCoAOMT10-GFP) used in the subcellular localization assay; (**B**) GFP, Bright, RFP, and Merge represents green fluorescence, light microscopy image, red fluorescence (H_2_B-mCheery, nuclear marker), and the combination of fluorescence signals and bright field, respectively. Scale bars = 50 μm.

To detect the distribution of StCCoAOMT10 in plant cells, the recombinant plasmid of StCCoAOMT10 fused with the green fluorescent protein (GFP) tag (35S::StCCoAOMT10-GFP) was infiltrated into tobacco leaves with nucleus-located mCherry (RFP). As shown in Figure 9, GFP fluorescence of the empty vector (35S::GFP) is located throughout the nucleus and cytoplasm, and the distribution pattern of StCCoAOMT10 was similar to the empty vector, implicating that StCCoAOMT10 might participate in the methylation of anthocyanins in the cytoplasm of the plant.

## 4. Discussion

O-methyltransferases (OMTs) are generally involved in the methylation modification of plant secondary metabolites, such as alkaloids, phenylpropanoids, lignins, and flavonoids [54,55]. OMT-mediated methylation enhances the bioactivity, stability, and solubility of particular natural compounds for adaptation and defense against environmental changes [56,57]. In recent years, the members and functions of OMT genes have been widely identified due to their importance in plant secondary metabolism [25,58]. The Caffeoyl-coenzyme A O-methyltransferase (CCoAOMT) family was classified as class I O-methyltransferase. CCoAOMT family members play a considerable role in catalyzing the O-methylation modification of different compounds in plants, including flavonoids, lignin, and phenylpropionic compounds. CCoAOMT was first isolated in 1989 from suspension cell cultures of *Petroselinum crispum* [59]. Subsequently, many CCoAOMT genes were identified from various plant species, including *P. trichocarpa* [60], *O. sativa* [41], *S. bicolor* [38], and *A. thaliana* [61]. For example, in Arabidopsis, seven CCoAOMTs were identified and classified into three groups. According to previous reports, CCoAOMT proteins can be divided into two categories: the *CCoAOMT* gene and *CCoAOMT*-like genes, of which the true *CCoAOMT* gene represents the orthologous gene with *AtCCoAOMT1* and *OsCCoAOMT1*, also named clade 1a, while *CCoAOMT*-like genes represent the remaining members, including clade 1b, clade 1c, and clade 2 [38,39]. In this study, a total of 12 *StCCoAOMT* genes were identified in *S. tuberosum* using bioinformatics methods and designated as *StCCoAOMT1*~*StCCoAOMT12*. All StCCoAOMT members were irregularly allocated to eight chromosomes. In addition, twelve StCCoAOMTs were clustered into three clades based on the phylogenetic relationship, and no StCCoAOMT members were clustered into the subbranch Ic, which is in compliance with that of Arabidopsis.

In Arabidopsis, *AtCCoAOMT1* was classified as clade Ia, which has been verified as a regulatory factor that participates in lignin biosynthesis [38,39,62]. At present, *StCCoAOMT1*~*5*, *StCCoAOMT11*, and *StCCoAOMT12* fall within the same branch as *AtCCoAOMT1*, suggesting these genes may have a potential function in lignin biosynthesis. *AtCCoAOMT6* was confirmed to participate in the biosynthesis of phenylpropanoid polyamine polymers in flower organs [63]. In addition, *AtCCoAOMT7* plays an important role in the biosynthesis of phenylpropane and flavonoid and shows an evident preference for the para methylation of dihydroflavonol and flavanone [63]. Thus, we speculate that *StCCoAOMT6*, *StCCoAOMT7*, *StCCoAOMT8*, and *StCCoAOMT10*, which have a closer phylogenetic relationship with *AtCCoAOMT7*, may be involved in flavonoid biosynthesis. Furthermore, *AtCCoAOMT7* also affects the ferulic acid content in cell walls by regulating S-adenosyl-L-homocysteine (SAH) degradation by binding with SAH hydrolase (SAHH) and S-adenosyl-L-methionine synthases (SAMS) [64].

Our analysis of motif composition and gene structure exhibited that the StCCoAOMTs shared similar motif organizations and intron–exon structures in the same clade, suggesting these genes remain relatively conserved during evolutionary processes. Meanwhile, some differences existed in StCCoAOMT members of different groups; for example, group Ia has the specific motif 8, indicating functional divergence among *StCCoAOMT* genes. Gene duplication plays a crucial role in gene evolution [65]. The plant CCoAOMT family experienced significant expansion during evolution, potentially to diversify the functions necessary to involve the biosynthesis of secondary metabolites and adapt to the environment. In this study, through collinearity analysis, we identified four pairs of tandem duplicated genes—*StCCoAOMT2*/*3*, *StCCoAOMT3*/*4*, *StCCoAOMT4*/*5*, and *StCCoAOMT7*/*8*—as well as two pairs of segmental duplicated genes—*StCCoAOMT1*/*11* and *StCCoAOMT1*/*12*—suggesting that tandem duplication events play an important role in expanding StCCoAOMT family members.

The *cis*-acting elements in the promoter region might act synergistically in accordance with their exclusive functions and growth and development stages, as well as specific conditions [66]. The AC element was generally presented in the promoters of numerous genes that participated in lignin biosynthesis, such as *PAL*, *C4H*, *CAD*, *CCR*, and *CCoAOMT*, indicating that the AC element plays crucial roles in the lignin biosynthetic pathway [67,68]. The MybPlant element in the gene promoter regions of the phenylpropanoid biosynthesis pathway controls lignin biosynthesis by associating with the P-box element [69]. Furthermore, the H-box element was also discovered in the promoters of genes associated with lignin biosynthesis [70]. The analysis of *cis*-regulatory elements found that lignin-related *cis*-elements were presented in the promoter domains of six *StCCoAOMT* genes (*StCCoAOMT1*, -*2*, -*7*, -*9*, -*10*, and -*11*), with most of them containing the P-box motif, suggesting these *StCCoAOMT* genes may be involved in lignin biosynthesis. A wide array of light-responsive elements was identified in the promoters of all *StCCoAOMTs* and hormone-responsive elements in *StCCoAOMT1*, *StCCoAOMT5*, *StCCoAOMT6*, *StCCoAOMT10*, and *StCCoAOMT11* (Figure 5), which may be associated with potato development and anthocyanin synthesis. In addition, several types of stress-responsive elements were also predicted in the *StCCoAOMT* gene promoters, including anoxic-, defense-, drought-, and low-temperature-responsive elements, indicating that the expression of *StCCoAOMTs* is possibly induced by anoxic stress, pathogen infection, drought, and low temperatures.

Flavonoids are a major group of secondary metabolites that are universally accumulated in plants and play extensive roles in phytohormone signaling, pigment accumulation, and defense against different environmental changes based on their various structures [57,71]. Several *CCoAOMT* genes from different plant species were characterized to display substrate preferences for flavonoids, especially anthocyanins [25,58]. In *V. vinifera*, a *CCoAOMT*-like gene was documented to be co-expressed with the methylated anthocyanins and red color in grape berries [72]. The transcript abundance of *AOMT1* was regulated by the MybA protein, and the variation of *AOMT2* structure determined the methylation level of anthocyanins in grapes [21]. Additionally, a CCoAOMT member confers a purple color for berry peels by controlling the methylation process of anthocyanins [20,73]. The *SsAOMT5* gene was reported to be involved in the methylation modification of anthocyanins in the peels of wax apples and had a critical effect on red coloration in wax apple peels [74]. However, there are few studies on the biological functionality involving *StCCoAOMT* genes in anthocyanin biosynthesis. Therefore, the transcript abundance of *StCCoAOMT* genes in potato tubers with different coloration was measured. The results showed that the expression levels of the majority of *StCCoAOMT* genes have no significant difference and only *StCCoAOMT10* has a significant expression in K149-P with purple tuber flesh, demonstrating that *StCCoAOMT10* was the primary factor influencing the methylation of anthocyanin. The overexpression or silencing of the *StCCoAOMT10* gene in potatoes should be conducted in the future to deeply understand the roles of the *StCCoAOMT10* gene.

Although the ultimate metabolites of the phenylpropanoid pathway, including anthocyanin biosynthesis, accumulate principally in the vacuoles, the pathways themselves are localized in the cytoplasm, causing speculation that enzymes that participate in the pathways are potentially distributed in the cytoplasm. Previous studies have revealed that the CsCCoAOMT1 protein from *Citrus reticulata* was localized in the cytoplasm and nucleus [27], while the VvCCoAOMT4 protein from *V. vinifera* was localized in the membrane and nucleus [25]. At present, the majority of StCCoAOMT proteins were predicted to be distributed in the cytoplasm by WoLF PSORT. To verify the subcellular localization of StCCoAOMTs, the fusion protein 35S::StCCoAOMT10-GFP was constructed and introduced into tobacco leaves. The result demonstrated that StCCoAOMT10 was a cytoplasmic and nuclear localization protein, indicating that StCCoAOMT10 might be involved in the methylation of anthocyanins in the cytoplasm of the plant.

## 5. Conclusions

**Figure 10:**
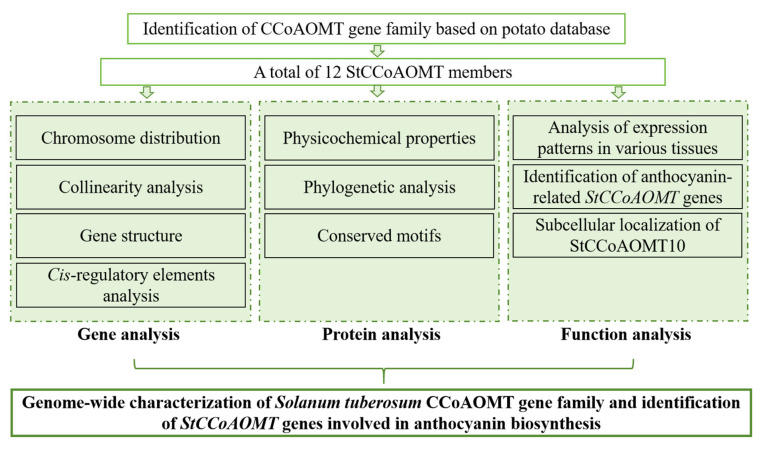
Schematic diagram of experimental design.

In conclusion, our work first identified the CCoAOMT family members in *S. tuberosum*. The genomic distribution, phylogenetic relationship, conserved motifs composition, and *cis*-regulatory elements of *StCCoAOMT* genes were characterized in detail. The transcript abundances of *StCCoAOMT* genes in various potato tissues were also investigated. In addition, the transcript levels of *StCCoAOMT10* had a significantly positive correlation with anthocyanin biosynthesis in potato tuber flesh. The subcellular localization assay displayed that the StCCoAOMT10 protein expressed in the cytoplasm and nucleus (Figure 10). This study provided useful information on the evolution of the CCoAOMT family in different plants and shed light on the potential roles of the *StCCoAOMT* genes in regulating potato anthocyanin biosynthesis.

## Figures and Tables

**Table 1:** Characterization of StCCoAOMT family members in potato.

Gene Name	Gene ID	Number of Amino Acids	Molecular Weight (kDa)	Theoretical pI	Instability Index	Aliphatic Index	GRAVY	Subcellular Localization
StCCoAOMT1	Soltu.DM.01G047320	248	27.99	5.53	37.62	101.45	−0.227	Cytoplasm
StCCoAOMT2	Soltu.DM.02G028520	242	27.33	5.29	42.1	97.15	−0.251	Cytoplasm
StCCoAOMT3	Soltu.DM.02G028530	175	19.69	5.18	40.75	109.26	−0.045	Cytoplasm
StCCoAOMT4	Soltu.DM.02G028540	242	27.25	5.29	39.05	97.15	−0.244	Cytoskeleton
StCCoAOMT5	Soltu.DM.02G028550	242	27.28	5.3	39.05	97.56	−0.245	Cytoskeleton
StCCoAOMT6	Soltu.DM.03G003400	441	49.72	5.13	42.69	106.37	−0.126	Cytoplasm
StCCoAOMT7	Soltu.DM.04G025040	244	27.57	5.3	37.84	98.73	−0.205	Cytoplasm
StCCoAOMT8	Soltu.DM.04G025050	282	31.76	5.32	46.16	95.11	−0.193	Chloroplast
StCCoAOMT9	Soltu.DM.08G001680	288	32.22	7.08	36.53	97.74	−0.106	Chloroplast
StCCoAOMT10	Soltu.DM.09G025040	234	26.33	5.51	35.93	103.33	−0.118	Cytoplasm
StCCoAOMT11	Soltu.DM.10G014940	245	27.85	5.14	34.38	100.73	−0.271	Cytoplasm
StCCoAOMT12	Soltu.DM.12G013090	123	13.72	4.93	55.56	111.71	−0.031	Cytoplasm

## Data Availability

The original contributions presented in the study are included in the article and Supplementary Materials, further inquiries can be directed to the corresponding author.

## Supplementary Materials

The following supporting information can be downloaded at https://www.mdpi.com/article/10.3390/genes15111466/s1, Figure S1: Conserved eight motifs of StCCoAOMT proteins; Table S1: Primers used in this study; Table S2. Tandem duplicated gene pairs of CCoAOMT gene family in potato; Table S3. The sequences of CCoAOMT protein from different species; Table S4. Syntenic gene pairs between potato and Arabidopsis, *C. sinensis*, tomato.
