# Supplementary figures and images for: Genome-Wide Characterization of Solanum tuberosum CCoAOMT Gene Family and Identification of StCCoAOMT Genes Involved in Anthocyanin Biosynthesis
Source: Genes (Basel). 2024 Nov 13;15(11):1466. doi: 10.3390/genes15111466 (PMC11593951; doi:10.3390/genes15111466)

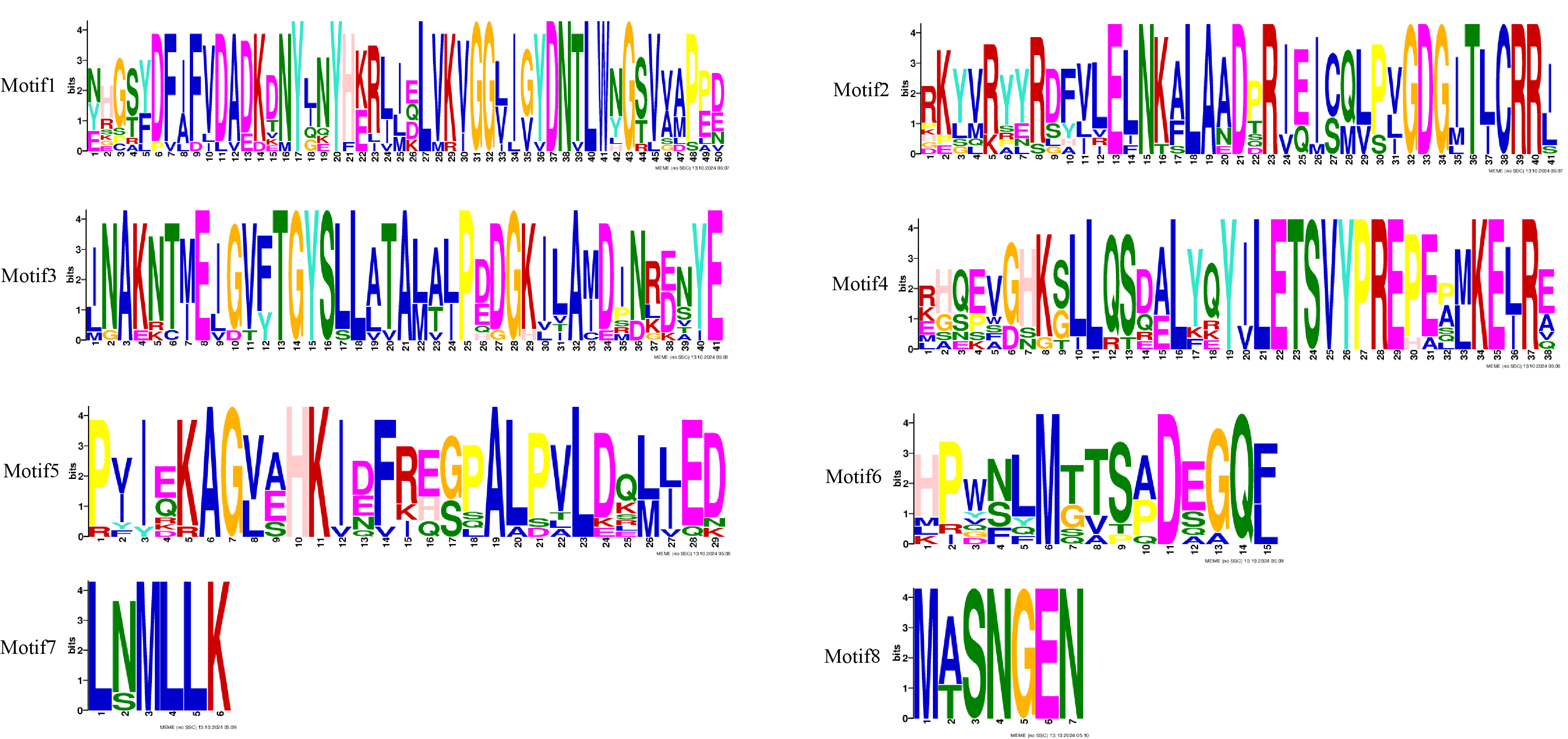

Supplement: Supplementary file 1 [file genes-15-01466-s001.zip › Figure S1.jpg]
